# Genetic Diversity of the Hepatitis C Virus Among Patients with HIV in EECA Countries

**DOI:** 10.3390/v18010016

**Published:** 2025-12-22

**Authors:** Vasiliy E. Ekushov, Maksim R. Halikov, Irina P. Osipova, Alexei V. Totmenin, Ludmila G. Gotfrid, Vardan G. Arzakanyan, Siranush V. Martoyan, Kristine V. Lalayan, Tamara V. Hovsepyan, Lilit H. Petrosyan, Susan G. Muradyan, Hermine M. Hovakimyan, Aibek A. Bekbolotov, Elmira B. Narmatova, Aida S. Karagulova, Kunduz T. Momushova, Aikanysh K. Djusupbekova, Baarinisa M. Iskanova, Aida K. Mamirbaeva, Ulukbek T. Motorov, Vitalla-Victoria V. Minikhanova, Sergey E. Skudarnov, Tatyana S. Ostapova, Alexander P. Agafonov, Natalya M. Gashnikova

**Affiliations:** 1 State Research Center of Virology and Biotechnology VECTOR, 630559 Koltsovo, Russia; halikov_mr@vector.nsc.ru (M.R.H.); osipova_ip@vector.nsc.ru (I.P.O.); gotfrid_lg@vector.nsc.ru (L.G.G.); agafonov@vector.nsc.ru (A.P.A.); ngash@vector.nsc.ru (N.M.G.); 2 National Center for Infectious Diseases, Ministry of Health, Yerevan 0041, Armenia; arzakanyanvardan@gmail.com (V.G.A.); smartoyan@gmail.com (S.V.M.); klalayan@rambler.ru (K.V.L.); thovsep@gmail.com (T.V.H.); lilitcom@yahoo.com (L.H.P.); suzy-1989@mail.ru (S.G.M.); hovakimyan80@mail.ru (H.M.H.); 3 Republican Center for Blood-Borne Viral Hepatitis and HIV Control, Bishkek 720017, Kyrgyzstan; aibek_0001@mail.ru (A.A.B.); elmira.narmatova@yandex.ru (E.B.N.); karagulova.ash@mail.ru (A.S.K.); m_kunduza@mail.ru (K.T.M.); aika081180@inbox.ru (A.K.D.); baarinisaiskanova@gmail.com (B.M.I.); mamyrbaeva_ak@mail.ru (A.K.M.); 4 Osh Regional Center of AIDS Treatment and Prevention, Osh 723500, Kyrgyzstan; umotorov@mail.ru; 5 Krasnoyarsk Regional Center for Prevention and Control of AIDS, 660017 Krasnoyarsk, Russia; minikhanova@aids.krsn.ru (V.-V.V.M.); gl_vrach@aids.krsn.ru (S.E.S.); ostapova@aids.krsn.ru (T.S.O.)

**Keywords:** HCV, subtypes, genetic diversity, EECA

## Abstract

Against the backdrop of active efforts to combat HCV worldwide with the help of DAAs, knowledge of the genetic diversity of HCV in the general population and in groups most at risk of infection is becoming increasingly important. The aim of this study was to characterize the molecular genetic diversity of HCV among individuals with HIV in Armenia, Kyrgyzstan and the Krasnoyarsk Krai region of Russia. The study included residents of Armenia (*n* = 73), Kyrgyzstan (*n* = 180) and the Krasnoyarsk Territory (*n* = 141) with HIV/HCV co-infection who were under observation at AIDS centers in these countries, collected between 2021 and 2023. The *Core/E1* gene fragments obtained were analyzed using the maximum likelihood method to create a phylogenetic tree. HCV subtype 3a was dominant in Armenia (56.2%) and Kyrgyzstan (51.4%). The circulation of HCV subtype 4a was detected for the first time in Armenia, while the spread of HCV genotype 2, represented by three different subtypes, was documented in Kyrgyzstan. The genetic diversity of HCV in Krasnoyarsk Krai is consistent with the findings of previous Russian studies. Phylogenetic analysis revealed the formation of HCV clusters with a high level of bootstrap support, suggesting shared transmission routes, predominantly among PWID. This suggests that there are common routes of HCV transmission between and within countries.

## 1. Introduction

The hepatitis C virus (HCV) is a leading cause of chronic liver disease worldwide. HCV infection can manifest as either an acute or chronic condition and may progress to liver cirrhosis and hepatocellular carcinoma (HCC) [1].

HCV is classified within the genus Hepacivirus of the Flaviviridae family. Its genome consists of a single-stranded, non-segmented, positive-sense RNA molecule approximately 9600 nucleotides in length, featuring a single long open reading frame that encodes a polyprotein of around 3000 amino acids. The gene order is as follows: *Core*/*E1*/*E2*/*p7*/*NS2*/*NS3*/*NS4A*/*NS4B*/*NS5A*/*NS5B* [2,3,4].

At its seventy-fifth session, the World Health Assembly approved a plan to combat viral hepatitis for the period 2022–2030. The WHO has set four targets for eliminating HCV as a public health threat by 2030, compared to baseline levels in 2015: diagnosing 90% of the population who have HCV, treating 80% of the population, reducing new HCV infections by 80%, and reducing HCV mortality by 65% [5]. However, the implementation of the WHO strategy is hindered by the extremely limited data on the prevalence, genetic diversity and level of drug resistance of HCV in EECA countries.

The prevalence of HCV infection in the WHO European Region varies significantly. In Northern, Western and Central European countries, HCV prevalence reaches 0.5%, whereas in many countries of Eastern Europe and Central Asia (EECA), it is as high as 5% [1].

Key populations play a pivotal role in the current epidemic. PWID represent the highest-risk group, exhibiting a high rate of HIV/HCV co-infection. Nearly half of HCV infections arise from unsafe injection practices among PWID [6]. Beyond parenteral transmission, substance use (not always injecting) may contribute to high-risk sexual behavior, increasing the likelihood of sexual HCV transmission. PWID are not only affected by the epidemic; they are also key indicators of active transmission. Studying this cohort offers a “sentinel lens” into the frontline of the epidemic.

Immunocompromised individuals are particularly vulnerable. Men who have sex with men (MSM) are another high-risk group, where widespread use of HIV-1 pre-exposure prophylaxis (PrEP) has been associated with rising HCV incidence. HCV prevalence among MSM ranges from 4% to 8% [7]. Notably, compared to MSM and individuals with sexually transmitted HCV, PWID are 2,5 times less likely to achieve a sustained virological response at 12 weeks (SVR_12_) [8].

The high mobility of EECA countries’ populations, as represented by labor migrants, also influences the overall nature of the epidemic and could result in the cross-border transmission of HCV. A systematic review of HCV prevalence among migrants worldwide showed that the prevalence of anti-HCV antibodies among migrants from EECA countries was around 2–3% [9].

In EECA countries, subtypes 1b and 3a are the most prevalent. The widespread circulation of subtype 3a in former Soviet Union states is linked to the expansion of injecting drug use in the 1960s and 1990s, whereas subtype 1b likely became established in the region due to historical blood transfusion practices [10,11]. Recent and comprehensive molecular epidemiology data for several EECA nations, including Armenia and Kyrgyzstan, remain scarce. This gap hinders the design of tailored prevention and treatment programs and limits understanding of cross-border transmission [10].

Treatment with direct-acting antivirals (DAAs) provides a key method of controlling HCV transmission. DAAs specifically target HCV proteins involved in viral replication, achieving SVR rates exceeding 90% [12]. Epidemic HCV subtypes served as in vitro models during DAA clinical trials, leading to the development of “pan-genotypic” DAAs effective against these subtypes [13]. However, treatment regimen selection and duration may be influenced by genotype/subtype (particularly for subtypes 1a, 1b and endemic variants), the presence of recombinant forms, cirrhosis and prior treatment failure [14,15]. Thus, HCV genotyping/subtyping is recommended before initiating DAA-based therapy [14,15]. Information on circulating HCV variants can also be used to track the cross-border transmission of the virus.

At present, a heterogeneous population of HCV circulates worldwide. According to the international nomenclature established by the ICTV, HCV is classified into 8 genotypes and 93 subtypes [16]. Additionally, there are reports of novel viruses not belonging to any previously described genotype/subtype, as well as recombinant viruses (e.g., 2k/1b) [16,17]. The distribution of genotypes/subtypes varies geographically. The most prevalent are subtypes 1a, 1b, 2a and 3a. These are termed “epidemic subtypes” and spread globally prior to the discovery of HCV in 1989 [10,13]. Other globally disseminated epidemic subtypes include 4a, 4d and 6a 13]. For many countries, data on HCV circulation is only available for the most common subtypes (1a, 1b and 3a), while the presence of other subtypes is often summarized under separate genotypes (GT2, GT4 and GT6) that are circulating in these countries. This can have a significant impact on HCV elimination strategies. HCV transmission primarily occurs through blood transfusions and/or blood products, iatrogenic procedures and injecting drug use [4,10,18]. The remaining HCV subtypes are considered “endemic,” with circulation restricted to specific geographic regions.

Detailed characterization of HCV subtypes within individual countries—as well as among key populations—could inform optimized strategies for HCV prevention, diagnosis and treatment under national viral hepatitis (VH) elimination programs aligned with the WHO’s goal of eliminating VH by 2030 [5].

This study aimed to characterize the molecular genetic diversity of HCV by analyzing *Core/E1* gene fragments among people living with HIV in Armenia and Kyrgyzstan, as well as residents of Russia’s Krasnoyarsk Krai.

## 2. Materials and Methods

### 2.1. Sample Source

The genetic diversity of HCV in the countries studied was assessed using a cohort observational study design. The patient sample was randomly selected from people living with HIV who had been diagnosed with HCV and regularly visited the AIDS Center for follow-up care and ART medication. This study aims to describe the genetic diversity of HCV circulating among individuals with HIV/HCV co-infection.

Inclusion criteria were individuals with HIV/HCV coinfection, aged 18 years or older, who are legally capable of providing informed consent to participate in the study.

Exclusion criteria were a lack of informed consent and the patient’s inability to understand the meaning and content of the project and answer questions.

The consecutive sample of patients was formed when they visited the relevant health center in their country.

The study cohort for assessing HCV genetic diversity comprised individuals with HIV/HCV co-infection from Armenia (*n* = 73), Kyrgyzstan (*n* = 179) and Russia’s Krasnoyarsk Krai (*n* = 141). AIDS Prevention and Control Centers in the respective countries and regions collected peripheral blood samples and clinical–epidemiological data from patients with HIV/HCV between 2021 and 2023. The Ethics Committee in each participating country approved the study protocols. All participants provided written informed consent, ensuring anonymized data usage in compliance with ethical guidelines. Clinical and epidemiological data included age, sex and probable HIV transmission route. Qualified specialists from the AIDS Center performed data collection after obtaining informed consent from the patient. Epidemiological data was collected through questionnaires that study participants completed when they visited a health center in their country. The questionnaire data was anonymized, and each patient was assigned a unique identification number. Patients were given the option to skip any questions in the questionnaire. Information about HIV infection (route of HIV transmission) was obtained during the standardized interview with the patient during the visit to the AIDS center.

### 2.2. HCV RNA Extraction, Amplification and Sequencing

HCV RNA was extracted from plasma in two stages using RIBO-zol-C and RIBO-prep kits (phenol–chloroform extraction method) (AmpliSens, InterLabService, Moscow, Russia), according to the manufacturer’s instructions (volume of 100 µL of plasma was used for extraction).

The two-step nested PCR with virus-specific primers was employed to amplify the target *Core/E1* region (~1000 bp).

To obtain *Core/E1* gene fragments, we used the BioMaster OT-PCR-Extra kit (Biolabmix, Novosibirsk, Russia) with thermostable reverse transcriptase (RNAscribe RT (genetically modified reverse transcriptase (RT) of mouse leukemia virus (M-MuLV)), HS-Taq DNA polymerase and Pfu DNA polymerase for the first round of PCR.

For the second round of PCR, we used the BioMaster HS-Taq PCR kit (Biolabmix, Novosibirsk, Russia), which contains highly process-intensive recombinant Taq DNA polymerase.

The reaction mixture was prepared according to the instructions included in the kit. Control samples (both negative and positive) that were prepared during the RNA extraction stage were used to monitor the quality of the reaction.

We estimated the length of the PCR products and the melting temperature of the primers using Oligo v.7 software (Molecular Biology Insights, Cascade, CO, USA). The reaction was performed in a T100 ThermalCycler (Bio-Rad, Hercules, CA, USA) and amplified under specified conditions (Supplementary Tables S1–S3).

For PCR product detection, horizontal electrophoresis was performed in 1% agarose gel with ethidium bromide. The «Step 50 plus» DNA marker (Biolabmix, Novosibirsk, Russia) was used for visual assessment of the PCR product. DNA fragments were visualized using a transilluminator (VilberLourmat, Collégien, France) with ultraviolet light at a wavelength of 312 nm.

Subsequent sequencing of HCV DNA amplicons was performed using the BigDye Terminator™ v3.1 kit on an Applied Biosystems 3500 Series Genetic Analyzer (Applied Biosystems, Foster City, CA, USA). The sequencing reaction was prepared following the current instructions.

The obtained data were analyzed using Sequencher 4.1 software (Gene Codes Corporation, Ann Arbor, MI, USA). The quality of each sequence was assessed by manually reviewing the chromatogram.

### 2.3. Genotyping

HCV RNA was extracted from plasma using phenol–chloroform extraction. A two-step nested PCR with virus-specific primers was employed to amplify the target *Core/E1* region (~1000 bp). Subsequent sequencing of HCV DNA amplicons was performed using the BigDye Terminator™ v3.1 kit on an Applied Biosystems 3130xl Genetic Analyzer (Applied Biosystems, Foster City, CA, USA).

The obtained HCV sequences were aligned with reference sequences of different subtypes and recombinant forms from the International Committee on Taxonomy of Viruses (ICTV) database using MEGA11 and AliView v1.30 [16,19,20]. Multiple sequence alignment was conducted with MAFFT v7.526 (RIMD) under default parameters [21].

For identification of closely related HCV strains, nucleotide sequences were analyzed using BLAST against the GenBank database (https://blast.ncbi.nlm.nih.gov/Blast.cgi (accessed on 5 March 2025)). Phylogenetic analysis of the *Core/E1* gene fragment (positions 368–1257 relative to H77) was performed via the maximum-likelihood method in IQ-TREE v1.6.12 [22], employing the GTR + I + G substitution model with 1000 bootstrap replicates to assess topology. The substitution model was chosen with IQ-TREE v1.6.12 (subsection: model selection). The resulting phylogenetic tree was visualized using iTOL v7 [23].

### 2.4. Data Availability

The sequences reported in this study are deposited in GenBank with the following accession numbers: PV754724 to PV754837 and OQ784306 to OQ784567.

## 3. Results

A total of 394 patients with HIV/hepatitis C virus (HCV) co-infection were enrolled in the study. The average patient age was 43 years, and 65% of patients were male. Liver cirrhosis was present in only a small percentage of the patients examined (2.0%; *n* = 8), which may be due to a lack of proper diagnosis. The proportion of migrant workers in the sample, especially from Kyrgyzstan, was lower than expected. This may be due to patients’ reluctance to share information about their time abroad.

Subtype 3a was the predominant type in all of the countries that were studied, except for Russia (Krasnoyarsk Krai), where subtypes 3a and 1b were equally prevalent. Armenia had the highest rate of subtype 3a.

As expected, the percentage of PWID in the study sample was high (56.1%; *n* = 221). Despite the widespread prevalence of subtype 3a in the PWID group (this subtype has become widespread in this risk group in the countries of the former USSR), subtype 1b was significantly more prevalent among PWID than expected. In Armenia, all patients with subtype 1b were PWID. In the Krasnoyarsk region of Russia, 81.0% of patients infected HCV with subtype 1b were PWID, compared to 71.0% for subtype 3a. In Kyrgyzstan, the prevalence of both subtypes among PWID was nearly equal: 3a (70.7%) and 1b (66.0%).

This is the first time that subtype 4a has been registered in Armenia. Previously, there had been no reports of this subtype circulating in the country. For the first time in the Kyrgyz Republic, GT2 was detected in circulation, represented by three subtypes: 2a, 2c and 2k.

### 3.1. Armenia

The mean age of patients was 44 years, with males predominating in the sample (90.4%) and females comprising only 9.6%. A history of injecting drug use (IDU) was reported by 52.1% of patients, all of whom were male; the specific types of drugs used were not specified. Labor migrants accounted for 20.5% of patients, with Russia (*n* = 12), Ukraine (*n* = 1) and Turkey (*n* = 1) being the main host countries; one labor migrant (LM) did not specify their migration destination. Individuals self-identifying as MSM comprised 4.1% of those tested, while one patient (1.4%) identified as transgender. Some 6.8% did not belong to any of the aforementioned key populations, and for 15.1% of patients, data on risk behaviors were unavailable (Table 1).

Among the study cohort, liver cirrhosis was reported in 8.2% of cases, with no cases of HCC observed. Of the patients with liver cirrhosis, three were infected with HCV subtype 3a, two with 1b and one with 1a.

Phylogenetic analysis of the *Core/E1* gene fragment revealed six HCV subtypes: 1a (15.1%; *n* = 11), 1b (20.5%; *n* = 15), 2a (1.4%; *n* = 1), 2k (5.5%; *n* = 4), 3a (56.2%; *n* = 41) and 4a (1.4%; *n* = 1) (Table 1).

Approximately half of all infections across subtypes occurred among PWID and their sexual partners (58.9%, *n* = 43). The highest proportion of PWID was observed for the epidemic subtype 3a (61.0%, *n* = 25). HCV subtypes 2k (*n* = 4), 1a (*n* = 11) and 1b (*n* = 15) were detected in both PWID and people living with HIV transmitted through sexual contact, with roughly equal distribution. Subtypes 1a and 4a were found exclusively in the PWID group.

**Figure 1:**
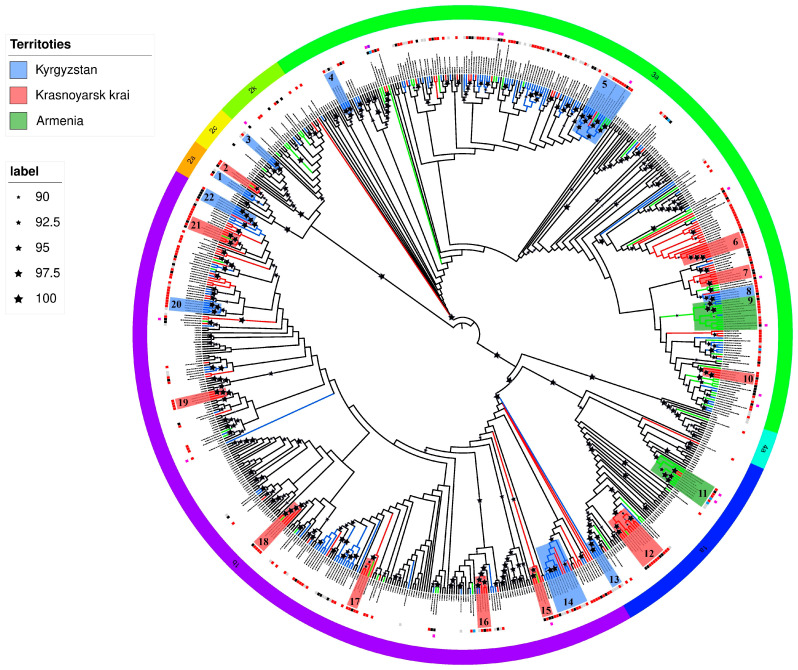
Maximum-likelihood phylogenetic tree of HCV *Core/E1* gene fragments. Black branches represent sequences from GenBank, red from Krasnoyarsk Krai, blue from Kyrgyzstan and green from Armenia. The color scheme of the clusters matches the colors of the branches, and each cluster is assigned a number (red—Krasnoyarsk Krai; blue—Kyrgyzstan; green—Armenia). The first outer ring indicates the risk group affiliation of sequenced patients: PWID—red; MSM—blue; outside risk groups—black; gray—no available data. The second outer ring marks additional risk groups: LM—pink; transgender—purple. The third ring shows subtype distribution.

Phylogenetic tree analysis revealed that HCV sequences from Armenia formed two distinct clusters (No. 9 and 11), suggesting a common transmission route among individuals with HIV/HCV co-infection (Figure 1).

One cluster (No. 9; bootstrap support value of 92) comprised HCV subtype 3a sequences obtained from ten patients (aged 41–61 years) at the Republican Center for Infectious Diseases of Armenia. Six patients were PWID, while two were presumably infected with HIV-1 through sexual contact (available data were insufficient to determine either the route of HCV transmission or the duration of infection), with one confirmed to have had a sexual partner who was a PWID. The cluster also contained HCV sequences from three male patients from Kyrgyzstan—two PWID and one presumably infected through sexual contact.

A second HCV cluster (No. 11; bootstrap support value of 98) contained six sequences of subtype 1a from the study cohort in Armenia. All viral variants in this group were isolated from men aged 23–48 years, containing two PWID, two MSM, one presumably infected with HIV-1 through sexual contact, and one with undetermined HIV-1 transmission route and/or risk group affiliation. The cluster also contained two HCV sequences from two male PWID (aged 34 and 39 years) from Krasnoyarsk.

The only case of HCV subtype 4a infection was identified in a 57-year-old male PWID residing in the Shirak region with no history of international travel.

A 32-year-old male PWID from Yerevan was found to be infected with HCV subtype 2a. Prior to the study, the patient had been diagnosed via qPCR with HCV subtype 3a but had not received treatment, with the estimated year of infection as 2009 (based on anamnestic data).

An additional HCV sample, previously subtyped as 3a (from a 48-year-old male PWID with an estimated infection date of 2019, based on anamnestic data), was reclassified as subtype 1a in this study.

### 3.2. Kyrgyzstan

HCV genotyping was performed among two groups. The first group comprised 112 consenting patients from the Republican Clinical Hospital for Infectious Diseases (Bishkek) and the Regional AIDS Center (Osh). The mean age was 45 years, with male predominance (83%) versus females (17%). PWID accounted for 67.9% of cases (93.4% male, 6.6% female); the types of drugs used were not specified. Two tested patients (1.9%) self-identified as MSM, one woman reported having sexual partners of both sexes, and 1.8% declined to answer. One patient was a labor migrant in Russia (Table 1).

Within this cohort, only one case (0.9%) of liver cirrhosis was reported (patient with HCV subtype 3a), with no cases of HCC identified.

The second group consisted of 68 completely anonymous blood samples collected at a diagnostic laboratory. Phylogenetic analysis of HCV *Core/E1* fragments from 180 sequences revealed six subtypes: 1a (2.8%; *n* = 5), 1b (41.1%; *n* = 74), 2a (2.2%; *n* = 4), 2c (1.7%; *n* = 3), 2k (1.1%; *n* = 2) and 3a (51.1%; *n* = 92) (Table 1).

The highest proportion of subtype 3a infections occurred among PWID (70.7%, n = 41), while 66.0% of patients with subtype 1b were also PWID. All patients with subtypes 1a and 2c were PWID, as was one of four patients with subtype 2a.

Phylogenetic analysis identified several statistically significant HCV sequence clusters (bootstrap support value > 90) which probably suggest common HIV/HCV transmission routes (Figure 1). Most clusters contained sequences from Kyrgyz PWID. For subtype 1b, three clusters (No. 14, 20 and 22; minimum bootstrap support 96) were identified: one predominantly comprising non-PWID patients, while the other two contained sequences from both PWID and patients not belonging to risk groups. Two clusters contained sequences from Russian (Krasnoyarsk Krai) residents.

Among three subtype 3a clusters (No. 4, 5 and 8; minimum bootstrap support 90), sequences from PWID predominated (except one cluster with predominantly missing epidemiological data). Sequences from Osh patients (mainly PWID) formed a distinct regional cluster (support 94). Only one cluster contained sequences from Russia (the Krasnoyarsk Krai) and Armenia (labor migrant with unspecified host country).

Among HCV subtype 1a sequences (No. 13), a cluster with bootstrap support value of 100 comprised four samples from Kyrgyzstan residents, with anamnestic data available only for one 40-year-old male PWID.

The single HCV subtype 2c cluster (No. 3; bootstrap support value of 100) contained viruses from three Kyrgyz patients—a 64-year-old male from the Chui region and two residents with no available anamnestic data.

Three HCV subtype 2a (No. 3) sequences from the Chui region residents—a 61-year-old male PWID, a 30-year-old woman presumably with HIV-1 transmitted through sexual contact, and one patient with missing anamnestic data—formed a cluster with a bootstrap support value of 94.

HCV subtype 2k was detected in two women with HIV women aged 40 and 45 years from Bishkek, both presumably infected through sexual contact, with one woman reporting sexual partners of both sexes.

### 3.3. Krasnoyarsk Krai

The mean age of patients included in the study was 41 years, with males predominating (68.8%) and females comprising 31.2% of samples. PWID accounted for 73.8% of cases, including 73.1% (n = 76) males and 26.9% (n = 28) females. One individual (0.7%) self-identified as a MSM and was also a PWID. Some 24.1% (n = 34) of patients did not belong to any risk group, with presumed HIV/HCV transmission occurring through heterosexual contact with individuals with HIV/HCV (Table 1).

Risk group data were unavailable for three individuals (2.1%). The cohort contained only one reported case of liver cirrhosis (patient with HCV subtype 3a), with no cases of HCC observed (though six non-HCC malignancies were recorded).

Phylogenetic analysis of *Core/E1* fragments identified five HCV subtypes: 1a (8.5%; *n* = 12), 1b (44.7%; *n* = 63), 2a (2.1%; *n* = 3), 2k (0.7%; *n* = 1) and 3a (44.0%; *n* = 62) (Table 1).

Most infections with epidemic subtypes occurred among PWID, with particularly high proportions for subtypes 1b (81.0%, *n* = 51), 3a (71.0%, *n* = 44) and 1a (58.3%, *n* = 7). Subtype 2k was exclusively found in PWID, while one of three (33.3%) subtype 2a cases occurred in a PWID.

The majority of Krasnoyarsk-derived HCV clusters comprised viruses from individuals sharing common HIV transmission routes, predominantly PWID. Six subtype 1b clusters (No. 15, 16, 17, 18, 19 and 21; minimum bootstrap support value of 96) were identified, with only one cluster dominated by non-PWID sequences. Four clusters contained HCV sequences from Armenian residents, containing both labor migrants (one being a Russian citizen from Tyumen) and non-migrants. One additional cluster contained two sequences from Kyrgyzstan residents (with unavailable anamnestic data).

Three subtype 3a clusters (No. 6, 7 and 10; minimum bootstrap support value of 91) consisted primarily of PWID-derived sequences and contained no international variants (Figure 1).

A distinct subtype 1a cluster (No. 12; bootstrap support value of 100) contained nine sequences from seven males and two females (aged 37–40 years), containing five male PWID and four individuals (two males, two females) presumably infected through sexual contact with PWID. This cluster also contained one sequence from a 47-year-old Armenian male PWID.

Two subtype 2a (No. 2) sequences formed a cluster, comprising one 41-year-old male PWID and one 46-year-old female infected through sexual contact. This cluster (bootstrap support value of 95) additionally contained a sequence from a 32-year-old Armenian male reportedly infected in Russia.

The sole Krasnoyarsk-derived subtype 2k sequence originated from a 46-year-old male PWID.

## 4. Discussion

Research on the genetic diversity of HCV strains circulating in Armenia has previously been limited. A 2016 study by Petruzziello et al. reported HCV genotype 3 prevalence at 37% [24], with genotypes 1 and 2 also detected. However, the study lacked fully sequenced HCV data, suggesting genotyping likely relied on qPCR. In a 2025 analysis by Mustafa et al., 18 publicly available sequences (NS3, NS5A and NS5B genes) were examined, predominantly subtype 1b (55.6%) and 3a (11.1%) [25]. This small sample size precludes robust assessment of subtype distribution across Armenia, compounded by the inclusion of sequences from Armenian migrants.

Our genotyping of 73 HCV variants from Armenia reveals striking disparities. Among patients with HIV/HCV co-infection, subtype 3a predominated (56.2%), while subtype 1b accounted for only 20.5%. Notably, ours is the first study to document subtype 4a infections in Armenia. As the patient infected with HCV subtype 4a had not traveled abroad, it is likely that he was infected domestically. This indicates that this subtype is spreading within the country.

Conventional genotyping employs qPCR targeting 5′-UTR and *Core* regions. Discrepancies emerged between our results and prior classifications by Armenia’s National Center for Infectious Diseases for two patients. One, initially genotyped as 3a, was reclassified as 2a based on our *Core/E1* sequence analysis; another, also previously labeled 3a, was identified as 1a. For the study, patients diagnosed with HCV had repeat blood samples taken. The first patient was a person who injects drugs (PWID), while the second had no known risk factors. Among PWID, reinfection with HIV or HCV—potentially altering viral dominance or generating recombinant strains—is not uncommon. However, full-genome sequencing is required to confirm recombination. Misclassification via qPCR or human error during diagnostic procedures cannot be ruled out. No other discrepancies in HCV genotyping were found. The test system correctly identified rare HCV genotypes, such as GT4 and GT2, in Armenia. However, qPCR does not involve classifying these rare genotypes into subtypes.

Two 2022 studies on individuals with HIV/HCV co-infection in Kyrgyzstan reported the following circulating HCV subtypes: 1a (2.6%), 1b (52.6%) and 3a (44.8%) [26]. Another study, analyzing publicly available HCV sequences, documented a different subtype distribution in Kyrgyzstan’s HCV population: 3a (53.7%) and 1b (44.7%) [25]. Our genotyping data show minor deviations from these earlier findings. Among the studied cohort with HIV/HCV co-infection, subtype 3a similarly predominated. Notably, we report for the first time the circulation of HCV genotype 2 in Kyrgyzstan, represented by three distinct subtypes. The presence of several GT2 subtypes in Kyrgyzstan suggests that there have been several independent cases of transmission.

Our data on HCV subtypes circulating among patients with HIV/HCV co-infection in the Krasnoyarsk Krai align broadly with prior Russian studies encompassing the general population with HCV, though with slight variations in the 3a/1b ratio. The proportion of subtype 3a in our cohort was marginally higher (40.2–42.1%) than values in the literature, while subtype 1b was somewhat lower (46.9%) [25,27]. These modest discrepancies may reflect regional epidemic dynamics in Krasnoyarsk or the specific profile of patients with HIV/HCV co-infection.

Collectively, our findings highlight the greatest HCV diversity in Armenia and Kyrgyzstan, where subtypes 4a and 2c were respectively detected. Although these genetic variants were rare, their identification in people living with HIV (PLWH) with no history of travel suggests localized transmission networks for these HCV strains. Our study also revealed divergent subtype distributions: in Russia and Kyrgyzstan, subtypes 1b and 3a dominate among PLWH—with near-equal prevalence in Russia—while 3a exceeds 1b by 10% in Kyrgyzstan. In Armenia, HCV 3a predominates (56.2%), alongside significant contributions from 1b (20.5%) and 1a (15.1%).

Patients with HIV/HCV co-infection reflect an “active epidemic”, in which transmission continues among IDUs (subtype 3a) and immunosuppressed patients. Earlier studies or studies of the general population reflect a “historical epidemic”, in which transmission primarily occurred through blood transfusions (subtype 1b). Our research suggests that the nature of the epidemic is changing within the PWID cohort. Specifically, there has been an increase in the number of PINs infected with HCV subtype 1b across all the countries studied.

Labor migrants contribute substantially to HCV transmission dynamics, potentially acquiring infection abroad or introducing strains into host countries. Significant migratory flows connect Russia, Kyrgyzstan and Armenia, with prolonged workforce mobility between these states. Since 2022, a marked increase in Russian migrants to Kyrgyzstan and Armenia has been observed, suggesting potential epidemiological linkages in HCV spread across these regions.

Six clusters were identified, combining sequences from Russia and Armenia for subtypes 1a, 1b and 2a. Two of the six clusters (1b No. 15 and 2a No. 2) had been confirmed to include patients from Armenia who had visited Russia. Reliable conclusions cannot be drawn about the cross-border transmission of HCV variants for the remaining clusters. However, this possibility cannot be ruled out, as patients could have been infected with an “imported” HCV variant within the country.

Similarly, five clusters were identified comprising sequences from Kyrgyzstan and the Krasnoyarsk Territory of Russia. However, the limited epidemiological data available for patients from Kyrgyzstan means we cannot confirm whether virus variants have spread from Kyrgyzstan to Russia, or whether citizens of Kyrgyzstan have become infected abroad.

The presence of a large number of independent clusters, each comprising sequences from different countries, may suggest the existence of a complex, interconnected meta-epidemic.

Since 2022, there has been a substantial increase in the number of Russians migrating to Kyrgyzstan and Armenia, suggesting potential epidemiological links in HCV transmission between these countries. Phylogenetic analysis revealed statistically significant regional clusters of HCV subtypes 1a, 1b and 3a with presumably shared HIV-1 transmission routes (though the precise HCV infection pathways remain uncertain due to a lack of seroconversion data). The overwhelming majority of individuals in these clusters were PWID. While definitive data on HCV infection timing and transmission modes are unavailable, these clusters likely represent HCV transmission through either injection drug use or sexual contact with PWID in the context of HIV-1-induced immunodeficiency.

The research has some limitations: 1. The sequential sampling design does not allow for a full characterization of the epidemic in the group with HIV/HCV infection. 2. There is a lack of information regarding the route and date of HCV infection in all countries. Many details in the survey (labor migration, route of infection) could have been concealed by patients despite the complete anonymity of the data. The disease was diagnosed primarily when patients visited AIDS centers, and, to a lesser extent, during annual medical exams. Determining the date of infection and the possible route of transmission is impossible in this regard. 3. For part of the Kyrgyzstani sample (68 patients), epidemiological data was unavailable. The samples were obtained from anonymous patients who only consented to participate in the HCV study on the conditions that they remained anonymous and were not required to answer questionnaires. 4. Analysis of the *Core/E1* region does not allow conclusions to be drawn about the presence of recombinant viruses.

## 5. Conclusions

The considerable genetic diversity and heterogeneous distribution of HCV variants necessitate further investigation into how specific genotypes/subtypes influence epidemic dynamics. Emerging evidence indicates reduced effectiveness of DAA therapies against endemic subtypes, which may potentially compromise HCV elimination efforts [12]. Targeted interventions focusing on key populations—PWID, MSM and sex workers—are crucial for effective HCV control. Comprehensive understanding of genotype/subtype distribution patterns should inform the development of national HCV prevention and treatment programs.

The EECA countries are experiencing a highly dynamic and interconnected HCV epidemic, characterized by active transmission among PWID. The high genetic diversity of HCV circulating in the region means that systematic molecular monitoring is not only recommended, but also necessary for public health. When developing and implementing HCV elimination programs, the constantly changing nature of the epidemic must be taken into account. This includes factors such as an increase in the proportion of patients infected with subtype 1b and active population migration.

This study was conducted among key populations at high risk of HCV infection and reflects epidemic patterns within vulnerable groups where PWID represent the predominant transmission drivers. The genetic diversity of HCV strains circulating in the general population may exhibit distinct characteristics.

The present work was undertaken as part of regional initiatives to reduce HCV transmission among people living with HIV in Eastern Europe and Central Asia. The findings provide critical evidence for developing long-term strategies to combat HCV effectively in the most vulnerable populations across participating countries, where the burden of HCV infection remains disproportionately high.

## Figures and Tables

**Table 1:** Baseline characteristics of patients with HIV/HCV co-infection.

	Armenia (*n* = 73)	Kyrgyzstan (*n* = 180)	Krasnoyarsk Krai (Russia) (*n* = 141)
Age, years	44	45	41
Sex			
Male	90.4% (*n* = 66)	51.7% (*n* = 93)	68.8% (*n* = 97)
Female	9.6% (*n* = 7)	10.6% (*n* = 19)	31.2% (*n* = 44)
No data	-	37.8% (*n* = 68)	-
Risk groups			
Not belonging to risks group	6.8% (*n* = 5)	16.7% (*n* = 30)	24.1% (*n* = 34)
PWID	52.1% (*n* = 38)	43.9% (*n* = 79)	73.8% (*n* = 104)
MSM	4.1% (*n* = 3)	1.1% (*n* = 2)	0.7% (*n* = 1)
LM	20.5% (*n* = 15)	0.6% (*n* = 1)	-
Transgender (without specifying a gender)	1.4% (*n* = 1)	-	-
nd	15.1% (*n* = 11)	38.3% (*n* = 69)	2.1% (*n* = 3)
Cirrhosis	8.2% (*n* = 6)	0.9% (*n* = 1)	0.7% (*n* = 1)
HCV subtypes			
1a	15.1% (*n* = 11)	2.8% (*n* = 5)	8.5% (*n* = 12)
1b	20.5% (*n* = 15)	41.1% (*n* = 74)	44.7% (*n* = 63)
2a	1.4% (*n* = 1)	2.2% (*n* = 4)	2.1% (*n* = 3)
2c	-	1.7% (*n* = 3)	-
2k	5.5% (*n* = 4)	1.1% (*n* = 2)	0.7% (*n* = 1)
3a	56.2% (*n* = 41)	51.1% (*n* = 92)	44.0% (*n* = 62)
4a	1.4% (*n* = 1)	-	-

## Data Availability

The original contributions presented in this study are included in the article/Supplementary Materials. Further inquiries can be directed to the corresponding author.

## Supplementary Materials

The following supporting information can be downloaded at https://www.mdpi.com/article/10.3390/v18010016/s1: Table S1: Primer sequences; Table S2: Protocol for the first round of PCR combined with RT; Table S3: Protocol for the second round of PCR.

## Abbreviations

The following abbreviations are used in this manuscript:

HIV	Human immunodeficiency viruses
HCV	Hepatitis C virus
EECA	Eastern Europe and Central Asia
DAAs	Direct-acting antivirals
AIDS	Acquired Immunodeficiency Syndrome
PWID	People who inject drugs
HCC	Hepatocellular carcinoma
ICTV	International Committee on Taxonomy of Viruses
MSM	Men who have sex with men
PrEP	Pre-exposure prophylaxis
SVR	Sustained virologic response
VH	Viral hepatitis
BLAST	Basic Local Alignment Search Tool
IDU	Injecting drug user
LM	Labor migrant
nd	No data
GT	Genotype
