# Supplementary material for: Genetic Diversity of the Hepatitis C Virus Among Patients with HIV in EECA Countries
Source: Viruses. 2025 Dec 22;18(1):16. doi: 10.3390/v18010016 (PMC12846408; doi:10.3390/v18010016)
Supplement: Supplementary file 1 [file viruses-18-00016-s001.zip › Table S1.pdf]

**Table S1.** Primer sequences

| Primers | Forward primer | Reverse primer |
|---|---|---|
| First PCR round | GGCCTTGTGGTACTGCCTGATAG | GGBGACCARTTCATCATCATRTCCC |
| Second PCR round | CTGATAGGGTGCTTGCGAGTG | TTCATCATCATRTCCCANGCCA |
