# Supplementary material for: Genetic Diversity of the Hepatitis C Virus Among Patients with HIV in EECA Countries
Source: Viruses. 2025 Dec 22;18(1):16. doi: 10.3390/v18010016 (PMC12846408; doi:10.3390/v18010016)
Supplement: Supplementary file 1 [file viruses-18-00016-s001.zip › Table S2.pdf]

Table S2. Protocol for the first round of PCR combined with RT.

<table>
<tr><th colspan="3">Thermocycling conditions</th></tr>
<tr><th>Temperature,°C</th><th>Time</th><th>Number of cycles</th></tr>
<tr><td>59</td><td>30 min</td><td>1</td></tr>
<tr><td>94</td><td>5 min</td><td>1</td></tr>
<tr><td>94</td><td>15 sec</td><td rowspan="3">10
Touchdown
t_{oa} -1°/cycle</td></tr>
<tr><td>t_{oa}+10°C</td><td>30 sec</td></tr>
<tr><td>68</td><td>0,5 min/kb</td></tr>
<tr><td>94</td><td>15 sec</td><td rowspan="3">20</td></tr>
<tr><td>$t_{оп}$</td><td>30 sec</td></tr>
<tr><td>68</td><td>0,5 min/kb</td></tr>
<tr><td>68</td><td>7 min</td><td>1</td></tr>
<tr><td>4</td><td>∞</td><td>∞</td></tr>
</table>

Abbreviations: t_{oa}: optimal annealing temperature for primers, kb: kilobase

The Touchdown method was used in the first 10 amplification cycles to increase the specificity of the amplified DNA fragments.
