# Supplementary material for: Genetic Diversity of the Hepatitis C Virus Among Patients with HIV in EECA Countries
Source: Viruses. 2025 Dec 22;18(1):16. doi: 10.3390/v18010016 (PMC12846408; doi:10.3390/v18010016)
Supplement: Supplementary file 1 [file viruses-18-00016-s001.zip › Table S3.pdf]

Table S3. Protocol for the second round of PCR.

Thermocycling conditions		
Temperature,°C	Time	Number of cycles
95	4 min	1
94	15 sec	10 Touchdown t_{oa} -1°/cycle
t_{oa}+10°C	30 sec	
72	1 min/kb	
94	15 sec	20
$t_{\text{оп}}$	30 sec	
72	1 min/kb	
72	7 min	1
4	∞	∞

Abbreviations: t_{oa}: optimal annealing temperature for primers, kb: kilobase

The Touchdown method was used in the first 10 amplification cycles to increase the specificity of the amplified DNA fragments.
